# Unraveling Anomalous Eutectic Formation in Ni-Sn Alloys During Directional Solidification with Transition Variable Speed

**DOI:** 10.3390/ma18214933

**Published:** 2025-10-28

**Authors:** Yongqing Cao, Huanhuan Cheng, Lianmei Song, Lei Wei, Lei Shi, Jiakang Li, Lixiao Jia, Miaoling Li, Derong Zhu

**Affiliations:** 1 Henan Key Laboratory of Green Building Materials Manufacturing and Intelligent Equipment, School of Intelligent Manufacturing, Luoyang Institute of Science and Technology, Luoyang 471023, China; suneycyq@163.com (Y.C.);; 2 Luoyang Ship Material Research Institute, Luoyang 471023, China; 3 School of Materials Science and Engineering, Luoyang Institute of Science and Technology, Luoyang 471023, China; 4 State Key Laboratory of Solidification Processing, School of Materials Science and Engineering, Northwestern Polytechnical University, Xi’an 710072, China

**Keywords:** eutectic solidification, anomalous eutectic, Ni-Sn alloy, directional solidification, velocity transition

## Abstract

This study investigates eutectic morphology transitions in Ni-Sn alloys using Bridgman directional solidification with a transition variable speed coupled with cellular automaton (CA) simulations. Steady-state solidification (0.1–2000 μm/s) produced only regular lamellar/rod-like eutectics, while velocity jumps triggered anomalous eutectic formation. As the drawing speed increased, the lamellar spacing decreased from ~3 μm to 0.4 μm, while the microhardness increased from ~426 HV to 500 HV. The experiments on Ni-Sn alloys revealed that anomalous eutectic morphologies form specifically at velocity transition interfaces (0.1–1000 μm/s), consistent with CA simulations showing destabilization of the lamellae, epitaxial growth of the Ni_3_Sn phase, and decoupled nucleation of the α-Ni phase for the formation. The work defines a processing window for anomalous eutectic formation and provides mechanistic insights bridging undercooling and directional solidification regimes.

## 1. Introduction

Eutectic solidification, a fundamental liquid–solid transformation, governs microstructural evolution in numerous industrial alloys and inorganic materials [1,2]. The regularity of eutectic morphology critically determines mechanical properties, particularly in self-reinforced composites where precise phase orientation is required [3]. Consequently, characterizing structural transitions between regular lamellar/rod eutectic and anomalous eutectic under non-equilibrium conditions remains central to solidification theory [4,5,6,7].

According to the Trivedi–Magnin–Kurz (TMK) model prediction [8], when the growth rate exceeds a certain critical value, the coupled growth of two phases becomes decoupled, resulting in anomalous eutectic. However, the application of the decoupled growth velocity predicted by the TMK model as the critical condition for anomalous eutectic formation remains controversial [9,10,11]. Li et al. [12] compiled critical undercooling values for anomalous eutectic formation in several alloy systems under deep undercooling conditions, revealing that even within the same alloy system—such as Ni-Sn eutectic—the reported critical undercooling for anomalous eutectic formation varies significantly among researchers, ranging from 10 K to 100 K. That is to say, the critical solidification conditions for the “regular eutectic–anomalous eutectic” transition remain uncertain.

For Ni-Sn alloys specifically, anomalous eutectic dominate in deep undercooling studies [13,14,15], where rapid recalescence generates ultrahigh cooling rates. However, this method intrinsically couples temperature gradient (*G*) and growth velocity (*V*), preventing independent control of these key parameters. This limitation obscures the individual roles of *G* and *V* in microstructural transitions, hindering mechanistic understanding of anomalous eutectic formation.

The Bridgman directional solidification process, as an effective method to study the microstructure of alloys and the relationship between their solidification parameters, can offer a solution by decoupling *G* and *V* during the solidification process [16,17]. Therefore, the accurate Bridgman directional solidification process may reveal the formation mechanism of anomalous eutectic, and can clarify the multi-factor influence of deep undercooling experiments [18,19]. This process explores the evolution of non-equilibrium solidification eutectic growth morphology [20]. For example, Cui et al. [21] prepared Fe-Al-Ta eutectic composites by a modified Bridgman directional solidification technique. Solidification microstructure transforms from regular eutectic to eutectic colony with the increase in the solidification rate. The solid–liquid interface of Fe-Al-Ta eutectic evolves from planar interface to cellular interface with the increase in the solidification rate. Corresponding, the existing Bridgman studies on Ni-Sn alloys also primarily focus on steady-state growth, reporting only regular lamellar/rod structures at different solidification rates [22]. This contrasts sharply with the ubiquitous anomalous eutectic in deeply undercooled Ni-Sn, suggesting a fundamental gap: under what kinetic conditions can anomalous eutectic form in directional solidification [23,24]. Recent work on Ag-Cu [25] suggests that thermal/diffusional instabilities—not impurities—trigger morphological transitions. The transition may require dynamic interface destabilization rather than steady-state growth. Nevertheless, systematic studies linking velocity perturbations to anomalous eutectic formation in Ni-Sn alloys remain absent.

Lin et al. [26] and Zhao et al. [27] both observed anomalous eutectic structures at the bottom of the molten pool in laser remelting/selective laser melting experiments on Ni-Sn near-eutectic alloys. Additionally, Requena et al. [28] also detected anomalous eutectic structures at the bottom of the molten pool in each cladding layer when investigating the microstructural evolution of Fe-Fe_2_Ti eutectic alloys during laser cladding. Nevertheless, anomalous eutectic structures are confined to a very small region at the bottom of the molten pool. Considering that the solidification process of the laser-remelted molten pool resembles directional solidification growth, within a certain temperature gradient range, the solidified microstructure grows from the bottom of the molten pool (with the growth rate approaching zero) to the top (with the growth rate approaching the laser scanning speed) at a varying growth rate. Anomalous eutectic structures can be observed because abrupt changes in the temperature field induce growth rate variations, both at the bottom of the laser-remelted molten pool and during the recalescence of deeply undercooled melts.

Wei et al. [29] employed the cellular automaton (CA) method to simulate the growth morphology evolution of Ni-Sn eutectic structures under transient variable-speed and constant-speed conditions based on directional solidification technology. The morphology of anomalous eutectic structures in the simulated Ni-Sn eutectic alloy under directional solidification with variable drawing speeds was highly similar to that observed in laser melting experiments. Requena et al. [28] used the phase-field method to simulate the morphology of anomalous eutectic structures at the bottom of Fe-Fe_2_Ti eutectic molten pools during laser cladding. However, the temperature gradient (*G*) and growth rate (*V*) adopted in the CA simulations by Wei et al. [29] and the phase-field simulations by Requena et al. [28] were estimated from the molten pool temperature field rather than precise experimentally measured values.

Recently, Zhang et al. [10] revealed the nucleation and growth modes upon rapid solidification of undercooled Co-24 at %wt.% Sn eutectic alloy. They pointed out that once an effective nucleus is formed to initiate crystallization, the solid–liquid interface will immediately migrate across the entire sample, being consistent with the as-solidified directional microstructure. Therefore, it is considered to study the formation mechanism of anomalous eutectic using the transient variable-speed directional solidification method.

To address this, Bridgman velocity-jump experiments are integrated with CA simulations. Using Ni-Sn alloys (hypoeutectic to hypereutectic), this work demonstrates that steady-state growth (0.1–2000 μm/s) preserves regular eutectic, confirming prior observations. Velocity transitions dynamically destabilize the solid–liquid interface, inducing localized anomalous eutectic. A distinct process window exists for anomalous eutectic formation, reconciling directional solidification with deep undercooling phenomena. This work establishes controlled velocity jumps as a novel pathway to probe non-equilibrium eutectic transitions, bridging the gap between classical solidification theory and rapid solidification regimes.

## 2. Materials and Methods

Ni-30 wt.%Sn (hypoeutectic), Ni-32.5 wt.%Sn (eutectic), and Ni-33 wt.%Sn (hypereutectic) alloys were prepared from commercial high-purity Ni (>99.99%) blocks and Sn (>99.99%) particles. The chemical compositions of the raw materials were tested by ICP-MS (Agilent 7900,Agilent Technologies, Santa Clara, California, USA ). As shown in Table 1 and Table 2, contents of all impurities in raw materials are less than 100 mg/kg (0.01%), meeting the application requirements. The raw materials were melted in a vacuum induction furnace (<10^−1^ Pa) and re-melted more than 3 times to homogenize the alloy composition. The chemical composition of the as-cast ingots were tested by EDS (X-MaxN 150, Oxford Instruments NanoAnalysis, High Wycombe, UK.). The results showed that the compositions of the Ni-Sn ingots are relatively consistent with the nominal components, as can be seen in Table 3. Then, the ingots were machined into Φ7 mm × 65 mm rods.

Bridgman directional solidification experiments used a vacuum Bridgman furnace with key capabilities: heating to 1400 °C, drawing speeds (0.1–2000 μm/s), and high vacuum (5 × 10^−4^ Pa). Experiment scheme for Ni-32.5 wt.%Sn eutectic alloy by Bridgman directional solidification is shown in both Table 4 and Table 5. Experiment schemes for Ni-30 wt.%Sn alloy and Ni-33 wt.%Sn alloy by Bridgman directional solidification are shown in Table 5. The directional solidification experiments were repeated three times.

Samples underwent preset velocity profiles, followed by quenching in ternary low melting point Ga-In-Sn liquid alloy with good thermal conductivity. Longitudinal sections were polished and etched (aqua regia, HNO_3_:HCl = 1:3). Microstructures were analyzed using Olympus GX71 optical microscopy and ZEISS ΣIGMA HD SEM. The Vickers hardness tests were conducted using a Duramin-A300 microhardness tester and loading 200 g for 10 s.

The CA modeled eutectic growth during velocity jumps (1 s transition from 0.1 μm/s to 1000 μm/s), with initial eutectic lamellar spacing λ = 0.5 μm, and *G* = 2.5 × 10^4^ K/m. The CA simulation parameters are as follows: Cell size: 0.01 μm; Domain size: X = 1024; Y = 10,240; Time step: 0.004 μs; Boundary conditions: the top and bottom are the no flux conditions; and the left and right are the periodic boundary conditions. For more information about the CA model for eutectic growth, see the reference [29].

## 3. Results and Discussion

### 3.1. Microstructural Morphology of Ni-Sn Alloys Under Different Drawing Speeds

#### 3.1.1. Ni-30 wt.%Sn Hypoeutectic Alloy

**Figure 1:**
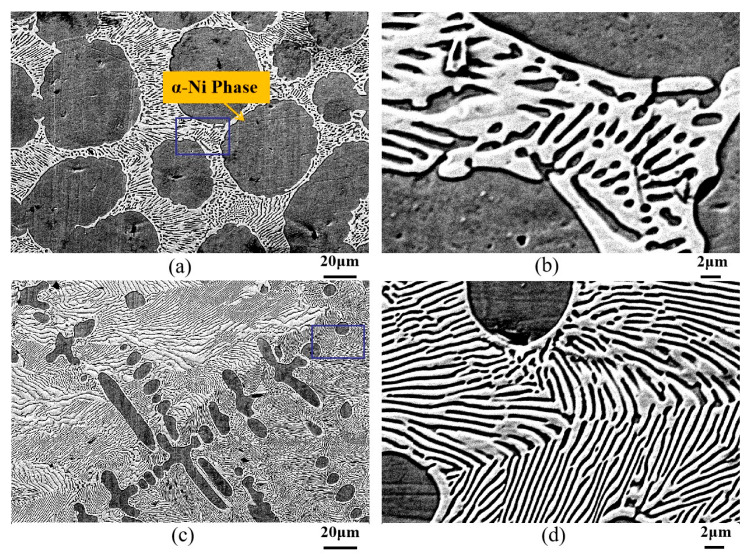
Microstructure of directionally solidified Ni-30 wt.%Sn hypoeutectic alloy at different drawing speeds. (**a**) Drawing speed 0.1 μm/s; (**c**) Drawing speed 1000 μm/s; (**b**,**d**) are the higher magnification images of rectangular zones in (**a**,**c**), respectively.

Figure 1 presents the microstructural morphology of the Ni-30 wt.%Sn hypoeutectic alloy solidified under Bridgman directional solidification conditions at various drawing speeds. Figure 1a depicts the microstructure at the initial drawing speed of 0.1 μm/s. Near-spherical black regions correspond to the primary α-Ni phase. Each primary α-Ni particle is enveloped by regular lamellar/rod-like eutectic, as can be seen in Figure 1b. Some primary α-Ni particles exhibit interconnection. Figure 1c shows the microstructure obtained at a constant drawing speed of 1000 μm/s. The primary phase adopts an equiaxed dendritic morphology. A thin white halo of Ni_3_Sn surrounds the primary α-Ni dendrites, while the interdimeric regions are filled with regular lamellar α-Ni + Ni_3_Sn eutectic, as can be seen in Figure 1d. Changes in the undercooling ahead of the solid–liquid interface during solidification critically influence crystallization. Generally, planar growth occurs in the absence of constitutional undercooling. The presence of constitutional undercooling induces a sequential transition in growth morphology: cellular → columnar → equiaxed.

According to the solidification principle [30], for morphological instability,

(1) $$G<-\frac{mV\Delta C0}{D}$$

where *G* is temperature gradient, *V* is the crystal growth rate, *m* is the liquidus slope, and *D* is the solute diffusion coefficient. Δ*C*_0_ = *C*_0_(1 − *k*)/*k,* where *C*_0_ is the composition of the alloy and *k* is the distribution coefficient. In the non-equilibrium solidification process, *k* is a function of *V.*

Because −mΔ*C*_0_ = Δ*T*_0_, the limit of constitutional undercooling can be expressed in its usual form:

(2) $$G/V=\Delta T_{0}/D$$

*G* and *V* are process variables and Δ*T*_0_ and *D* are alloy properties. Instability will result if *G/V* is smaller than Δ*T*_0_*/D*.

For the selected alloy, *G* and *V* are the main parameters determining the morphology and scale of the solidified microstructure. If the heat flow is unidirectional, the *G·V* product can be used to estimate the cooling rate, which in turn allows estimation of the microstructural scale; while the value of *G/V* determines the growth morphology of the microstructure [30]. During directional solidification, the drawing speed is equivalent to the crystal growth rate. As the drawing speed increases, the value of *G/V* decreases. The interface progressively destabilizes and the microstructure gradually alters.

As the drawing speed increases, the volume fraction of the higher-melting-point α-Ni phase increases, leading to solute enrichment (primarily lower-melting-point Ni_3_Sn) in the liquid. This enrichment lowers the actual crystallization temperature at the interface, initiating constitutional undercooling. The onset of constitutional undercooling promotes the formation of α-Ni as cellular crystals. With further increases in drawing speed, the α-Ni phase develops a columnar dendritic morphology, ultimately transitioning to an equiaxed dendritic structure.

#### 3.1.2. Ni-32.5 wt.%Sn Eutectic Alloy

**Figure 2:**
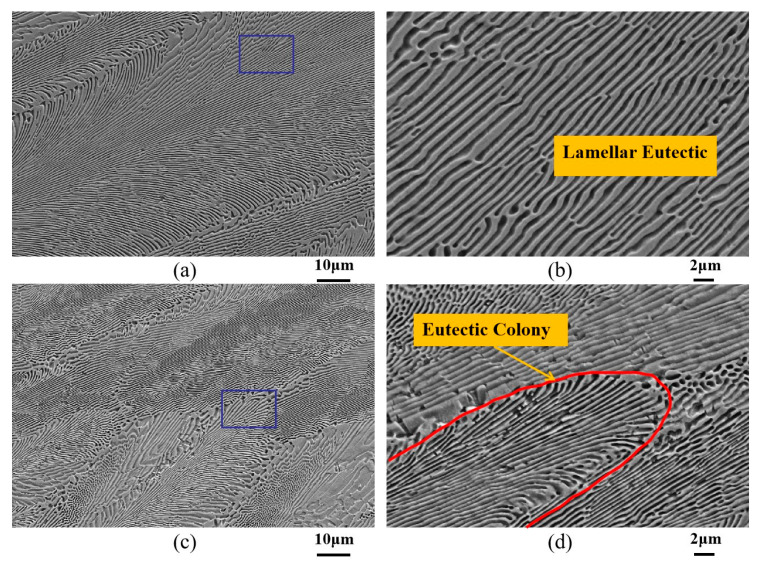
Microstructure of directionally solidified Ni-32.5 wt.%Sn eutectic alloy at different drawing speeds. (**a**) Drawing speed 1000 μm/s; (**c**) Drawing speed 2000 μm/s; (**b**,**d**) are the higher magnification images of rectangular zones in (**a**) and (**c**), respectively.

Figure 2 illustrates the microstructural morphology of Ni-32.5 wt.%Sn eutectic alloy directionally solidified using the Bridgman technique at different drawing speeds. As shown in Figure 2a, under a constant drawing speed of 1000 μm/s, the microstructure consists of regular lamellae. The eutectic lamellar spacing is approximately 0.9 μm, and a high magnification picture is shown in Figure 2b. Increasing the drawing speed to 2000 μm/s results in a more complex morphology due to differing eutectic colony orientations; however, the overall eutectic structure remains lamellar, as evidenced in Figure 2c. The eutectic lamellar spacing is approximately 0.5 μm, as shown in Figure 2d. As the solidification rate increases, the eutectic lamellar spacing gradually decreases. Notably, under steady-state constant drawing speeds, the Bridgman directionally solidified Ni-Sn eutectic alloy did not exhibit anomalous eutectic structures similar to those observed in deeply undercooled or laser-remelted Ni-Sn alloys.

The formation of eutectic colonies has been extensively studied. The impurity elements significantly influence morphological transitions in eutectic alloys. Impurity elements can induce a substantial region of constitutional undercooling ahead of the solid–liquid interface [31]. The resulting solute concentration gradient that is perpendicular to the interface destabilizes the planar interface, promoting a transition to cellular growth and the formation of eutectic colonies/cells. Yamauchi [32] demonstrated that minor additions of Mn and Co elements can alter the microstructure of directionally solidified Fe-Si eutectic alloys. Furthermore, severe segregation of a third element combined with a low ratio of *G* to *V* can even lead to dendritic eutectic structures.

Zhao et al. [33] observed cellular eutectic during their studies on undercooled solidification of Ag-Cu eutectic alloys. They emphasized that high-purity starting materials were used, effectively ruling out impurity-induced cellular interface formation. They proposed that the large difference in composition between the two eutectic phases and the very large thermal diffusion coefficient of the liquid should be responsible for the cellular growth of lamellar eutectic at low undercoolings. Tang et al. [34] pointed out that during the growth of a eutectic alloy, an increase in the growth velocity will trigger instability of the solid–liquid interface because of the occurrence of constitutional undercooling in front of the interface, which can lead to eutectic cells.

Recently, Kang et al. [22] studied the influence of impurity Nb on the microstructure of Ni-Sn and Co-Sn alloys during directional solidification. They pointed out that in the directional solidification of (Co_76_Sn_24_)_99.5_Nb_0.5_ eutectic alloy, the eutectic interface transits by planar–cellular–dendritic–seaweed morphology with increasing withdrawal velocity (*V*) at the temperature gradient (*G*) of 200 K/cm. When *G* increases to 300 K/cm, the cellular interface changes into the seaweed interface. The experiment showed that in addition to the impurity elements, process variables *G* and *V* also affects the microstructure morphology.

Campo et al. [23] researched the microstructures of directional solidification of the Al0.8CrFeNi2.2 eutectic high-entropy alloy. They found that regular lamellar eutectic became eutectic cells surrounded by intercellular regions as the translation rate increased to 8.4 and 14 μm/s. They believed that the solid–liquid interface becomes unstable for the increase in translation rates, and a transition from planar to cellular growth takes place.

In our study, high-purity Ni and Sn were used for melting alloys and ICP analysis, effectively excluding impurity influences. This phenomenon can be attributed to the increase in the solidification control parameter *V*, which reduces *G/V* and consequently affects the stability of the eutectic growth interface. With increasing solidification velocity, the undulations of the eutectic interface intensify, and the amplitude of fluctuations increases. When the amplitude reaches a certain level, it disrupts the coupled eutectic growth of the two phases, causing some eutectic phases to protrude and grow preferentially. Within these cellular or dendritically growing eutectic phases, the size of one single phase increases. Concurrently, certain eutectic phases experience growth depressions; these phases will either be gradually eliminated during continued growth or attach to the edges of the protruding eutectic phases and form eutectic colonies.

#### 3.1.3. Ni-33 wt.%Sn Hypereutectic Alloy

**Figure 3:**
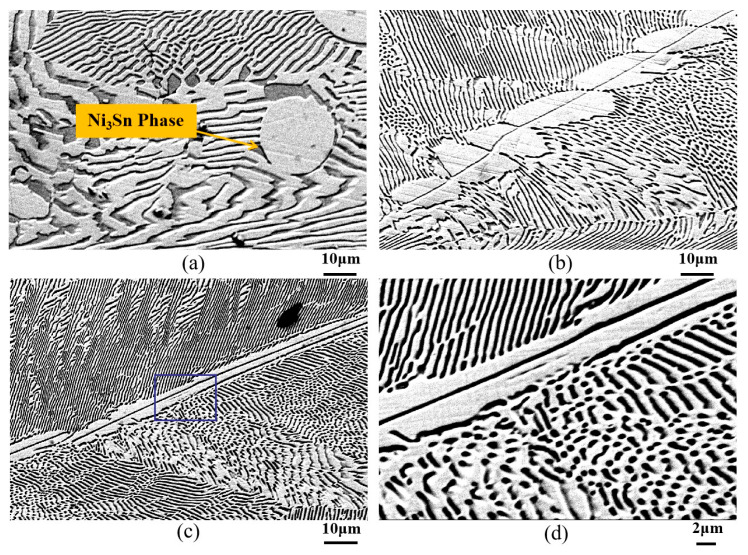
Microstructure of directionally solidified Ni-33 wt.%Sn hypereutectic alloy at different drawing speeds. (**a**) Drawing speed 0.1 μm/s; (**b**) Drawing speed 500 μm/s; (**c**) Drawing speed 1000 μm/s; (**d**) is the higher magnification image of rectangular zone in (**c**).

The microstructural morphology of directionally solidified Ni-33 wt.%Sn hypereutectic alloy at different drawing speeds is shown in Figure 3. During solidification of the hypereutectic Ni-33 wt.%Sn alloy, the primary Ni_3_Sn phase precipitates first. The near-spherical white structures in Figure 3a represent the primary Ni_3_Sn phase. However, increased in drawing speed significantly alters the morphology of the primary Ni_3_Sn phase. Figure 3b shows the microstructure resulting from a drawing speed of 500 μm/s. Here, the white “dendrite trunks” are composed of interconnected near-spherical primary Ni_3_Sn particles. Lamellar and rod-like eutectic structures grow epitaxially along these trunks, exhibiting specific orientations. When the drawing speed increased to 1000 μm/s, the trunk morphology gradually evolved towards a linear form, as seen in Figure 3c. The microstructure is similar to the feathery lamellar eutectic grain when the Ni-32.5 wt.%Sn eutectic alloy was undercooled below 100 K. Wei et al. believed that the alternating α-Ni and Ni_3_Sn lamellae both originated from a primary Ni_3_Sn twin plate [35].

Statistical analysis revealed that with the increase in drawing speed, the width of the primary Ni_3_Sn phase decreased progressively from an initial 28 μm to 20 μm, and ultimately to a linear structure of approximately 7 μm. The eutectic lamellar spacing λ decreased from an initial value of approximately 3 μm to 1.5 μm, and ultimately reached 0.5 μm when the drawing speed was 1000 μm/s, as shown in Table 6.

According to the solidification principle [30], the *G·V* product can be used to estimate the cooling rate, which in turn allows estimation of the microstructural scale. As the drawing speed increases, the characteristic size of the solidification microstructure decreases. As the drawing speed increases, the characteristic size of the solidification microstructure decreases.

Jackson and Hunt [36] established the classic steady-state non-faceted/non-faceted regular eutectic growth model by extending their analysis of steady-state diffusion fields to incorporate both diffusion effects and interfacial energy. This model, developed under low growth rates, solves the quasi-steady-state diffusion field equation to derive the solute distribution ahead of the liquid–solid interface front during coupled growth. It reveals that

(3) $$\lambda^{2}V=C$$

where *λ* is the eutectic spacing, *V* is the growth rate, and *C* is a constant.

Therefore, a change in the drawing speed affects the eutectic growth, particularly the solute distribution near the solid–liquid interface, which also changes the melt undercooling that leads to adjustment of the eutectic spacing to maintain growth near the extremum condition (minimal undercooling) [23,36].

Under a constant drawing speed of 1000 μm/s, the eutectic structure tended towards regular lamellar/rod-like morphology, with no anomalous eutectic observed. In conclusion, as the drawing speed increased, the characteristic scale of the microstructure gradually became finer.

### 3.2. Effect of Transition Variable Speeds on Ni-Sn Eutectic Microstructural Morphology

#### 3.2.1. Microstructural Morphology at the Transition Interface in Ni-30 wt.%Sn Hypoeutectic Alloy

**Figure 4:**
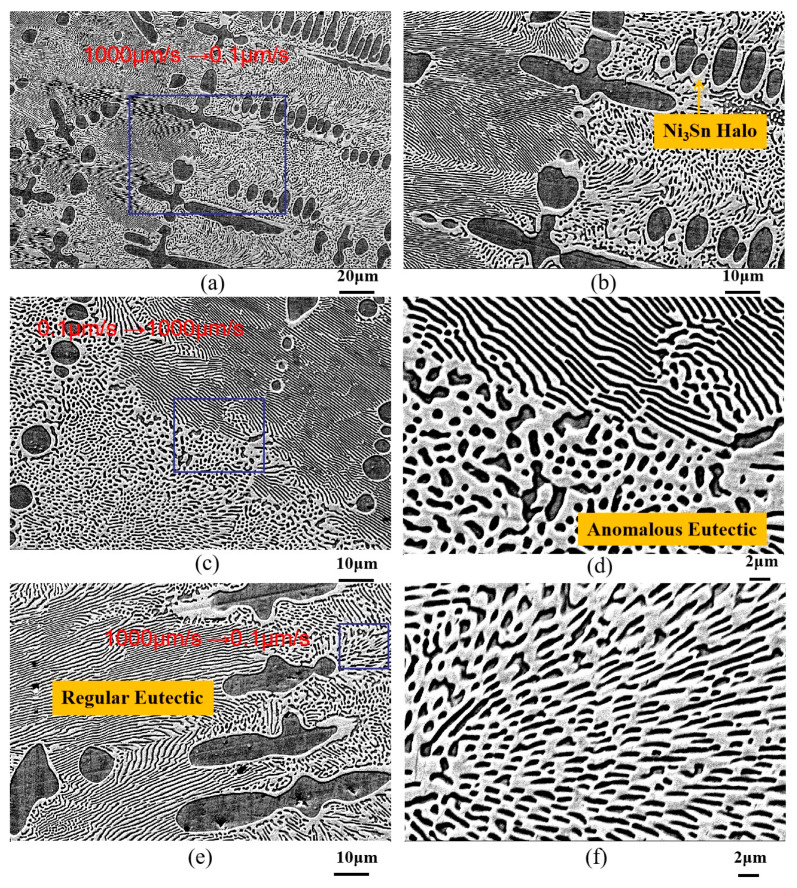
Microstructure evolution of Ni-30 wt.%Sn hypoeutectic alloy during directional solidification with transition variable speed between 0.1 μm/s and 1000 μm/s: (**a**,**c**,**e**) are the different transition interfaces; (**b**), (**d**), and (**f**) are the higher magnification images of rectangular zones in (**a**,**c**,**e**), respectively.

Figure 4 depicts the microstructure evolution of Ni-30 wt.%Sn hypoeutectic alloy during directional solidification with a transition variable speed between 0.1 μm/s and 1000 μm/s. Figure 4a shows the interface region, where primary α-Ni dendrites transition from fine to coarse, accompanied by a significant increase in primary dendritic arm spacing. The primary dendritic arm spacing changed approximately from 40 μm to 55 μm. Figure 4b reveals that the interdimeric eutectic structure transforms from regular lamellar to rod-like morphology. This indicates a jump from a high to a low growth rate at this interface. That is to say, the drawing speed jumped from 1000 μm/s to 0.1 μm/s.

Additionally, Ni_3_Sn halos constantly form around the primary α-Ni dendrites, as shown in Figure 4b. Halos of a second phase around the primary phase often form in the solidification of off-eutectic alloys, but the underlying mechanism has not been clarified. Recently, Qin et al. [37] proposed that halos form merely if the second phase can wet the primary phase based on the findings in Ag–Cu and Ni-Sn alloys. They believed that the halo formation is independent of the solidification mode, regardless of free or directional solidification, as well as how large the melt undercooling prior to nucleation is.

Figure 4c shows another interface formed by a drawing speed jump from 0.1 μm/s to 1000 μm/s. The left side exhibits rod-like eutectic, while the right side displays regular lamellar eutectic. Crucially, at the boundary between the rod-like and lamellar eutectic regions (lower left corner of Figure 4d), a small amount of anomalous eutectic structure, similar to that in deeply undercooled Ni-Sn alloys, is observed. The area fraction of anomalous eutectic is approximately 30% (Figure 4a). With a further jump in drawing speed from 1000 μm/s to 0.1 μm/s, the microstructure evolves as shown in Figure 4e, where regular lamellar eutectic transforms into anomalous eutectic and short rod-like structures (high magnification in Figure 4f). This suggests that under steady-state directional solidification, regular lamellar eutectic is the preferred growth morphology. During a jump speed, interface instability causes the regular lamellar structure to first transform into anomalous eutectic, subsequently evolving into a short rod-like morphology. From the experiment, it can be found that the eutectic morphology undergoes a transition following the sequence: “regular lamellar eutectic → anomalous eutectic + rod-like eutectic → regular lamellar eutectic” with the abrupt change in the drawing speed of the directional solidified Ni-30 wt.%Sn hypoeutectic alloy.

#### 3.2.2. Microstructural Morphology at the Transition Interface in Ni-32.5 wt.%Sn Eutectic Alloy

**Figure 5:**
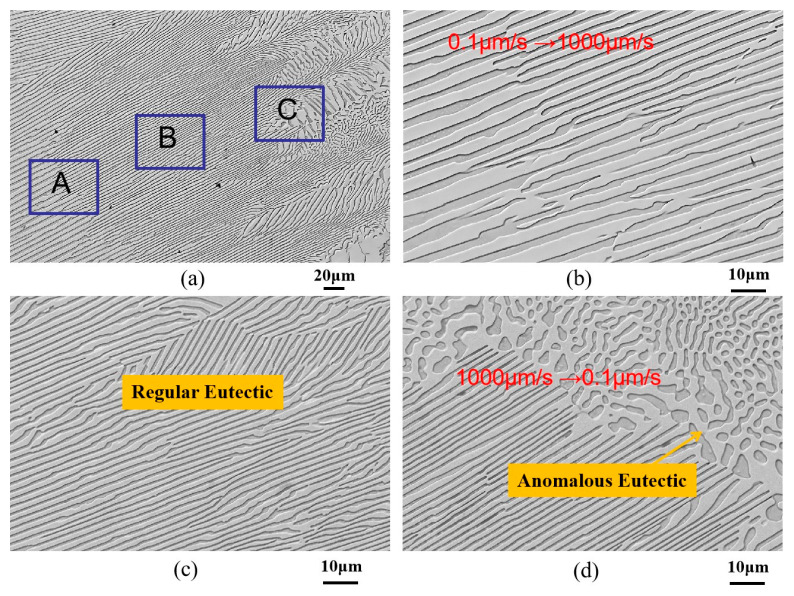
Microstructure evolution of Ni-32.5 wt.%Sn eutectic alloy during directional solidification with transition variable speed between 0.1 μm/s and 1000 μm/s: (**a**) the transition interface; (**b**–**d**) are the higher magnification images of regions A, B, and C shown in Figure 5a, respectively.

Figure 5 presents the microstructure evolution of an Ni-32.5 wt.%Sn eutectic alloy during directional solidification with a transition variable speed between 0.1 μm/s and 1000 μm/s. Figure 5a shows the transition interface, clearly divided into three distinct regions (A, B, and C) based on morphology. Higher magnification images of regions A, B, and C are shown in Figure 5b, c, and d, respectively. Following the rate jump, the initially coarse regular lamellar eutectic progressively refines into a fine regular lamellar structure with reduced lamellar spacing (Figure 5b). Due to the slight sample tilt during observation, the lamellar growth direction appears at an angle to the longitudinal axis of the specimen cross-section. Quantitative analysis confirmed that the eutectic lamellar spacing decreased from 3.8 μm to approximately 2.5 μm. Remarkably, this refined spacing (~2 μm) persisted even during the subsequent constant velocity pulling at 1000 μm/s after the jump. This refinement mechanism is attributed to the sudden increase in solidification velocity during the jump. Solute in the liquid ahead of the interface cannot diffuse laterally fast enough, leading to solute pile-up near the interface [38]. For the eutectic composition alloy (Ni-32.5 wt.%Sn), the initial precipitation of the α-Ni phase during solidification enriches the adjacent liquid in the Ni_3_Sn. This increased solute concentration causes lamellar depression. When this depression reaches a critical point, a new phase nucleates within the depression, and the other phase branches via a “bridging” mechanism, effectively reducing the lamellar spacing [39].

With further jumps in drawing speed, the growth direction of the lamellar eutectic shifts, resulting in an angle between the lamellae and the solidification (withdrawal) direction (Figure 5c). Crucially, at another jump transition zone, a transformation from regular lamellar eutectic to anomalous eutectic was observed at the solidification front, as indicated by the rectangle in Figure 5d. The morphology of this anomalous eutectic resembles that found at the bottom of laser-remelted Ni-Sn eutectic alloy samples [26] and undercooled solidification [13,14,15]. The area fraction of anomalous eutectic is approximately 10% (Figure 5a). This demonstrates that abrupt changes in solidification velocity during directional solidification can induce anomalous eutectic formation.

From the experiment, it can be found that the eutectic morphology undergoes a transition following the sequence “regular coarse lamellar eutectic→ fine lamellar eutectic → anomalous eutectic + rod-like eutectic → regular coarse lamellar eutectic” with the abrupt change in the drawing speed of the directional solidified Ni-32.5 wt.%Sn eutectic alloy.

Additionally, when drawing the Ni-32.5 wt.%Sn eutectic alloy at a constant speed of 1000 μm/s, the eutectic lamellar spacing is approximately 0.9 μm (Figure 2b); when the drawing speed changes from 0.1 μm/s to 1000 μm/s, the eutectic lamellar spacing is approximately 2 μm (Figure 5d). Furthermore, the lamellar eutectic after the transient speed change is more regular than that under a constant drawing speed. This phenomenon may be attributed to history-dependent selection [40].

Kang et al. [22] pointed out that eutectic growth is history-related, i.e., the eutectic morphology formed after changing the drawing speed is influenced by the preceding eutectic. Although the (Co_76_Sn_24_)_99.5_Nb_0.5_ eutectic alloy solidified into eutectic cells and then seaweeds when the drawing speed of 3 μm/s was abruptly changed to 10 and 80 μm/s, respectively, the obtained eutectic lamellae are more regular than those solidified at a constant drawing speed. Additionally, no anomalous eutectic were observed during sudden speed transitions in their research. However, in undercooling solidification, anomalous eutectic are very commonly present in Co-Sn and Ni-Sn systems.

The comparison between anomalous eutectic under deep undercooling and these formed under directional solidification conditions are as follows:

Thermodynamic factors dominant the anomalous eutectic formed in undercooled solidification. By drastically increasing undercooling to alter the thermodynamic driving force for nucleation and growth, the core mechanism lies in suppressing primary phase nucleation while promoting eutectic phase nucleation and growth at high undercooling and remelting during the recalescence period [41].

Kinetic factors dominate the anomalous eutectic formed in directional solidification with a transition variable speed. Through abrupt growth rate transitions modify interface stability, the core mechanism involves kinetic conditions (constitutional undercooling and interface instability) driving eutectic phase nucleation and growth within non-equilibrium solute-enriched zones. This process exhibits no direct correlation with thermodynamic undercooling (bulk melt undercooling).

However, anomalous eutectic was not observed at all jump transition interfaces, indicating that its formation occurs within a specific processing window.

The following should be noted based on our experiments using directional solidification of the Ni-32.5 wt.%Sn eutectic alloy: When tested according to the process parameters in Table 1, a speed within the range of 0.1 μm/s to 2000 μm/s resulted in no anomalous eutectic formation in the specimen. When tested according to the process parameters in Table 2, changing the drawing speed within the range of 0.1 μm/s to 1000 μm/s resulted in anomalous eutectic. This implies the formation of anomalous eutectic is associated with the magnitude of the speed change (ΔV). In our experiments, ΔV is approximately 1000 μm/s.

Furthermore, the acceleration rate of the speed change (the rate of change over time, acceleration a = ΔV/Δt) is also critically important. In our experiments, Δt is approximately 1 s. For the Ni-30 wt.%Sn hypoeutectic alloy and Ni-32.5 wt.%Sn eutectic alloy, anomalous eutectic formed under deceleration conditions (from 1000 μm/s to 0.1 μm/s within 1 s). The processing window for generating anomalous eutectic via step-changes in speed is very narrow, and the amount of anomalous eutectic is very small.

A large acceleration implies that the solidification rate undergoes drastic changes within an extremely short time. This strongly perturbs solute diffusion and heat flow conditions ahead of the solid–liquid interface, potentially leading to localized rapid cooling, intensified constitutional supercooling, interface growth instability, and other effects, thereby inducing the formation of anomalous eutectic. However, the parameter range (processing window) required to achieve this specific combination of transition magnitude and acceleration is very narrow. This explains why the amount of anomalous eutectic formed is typically small and why there are substantial challenges in process control.

#### 3.2.3. Microstructural Morphology at Transition Interfaces in Ni-33 wt.%Sn Hypereutectic Alloy

**Figure 6:**
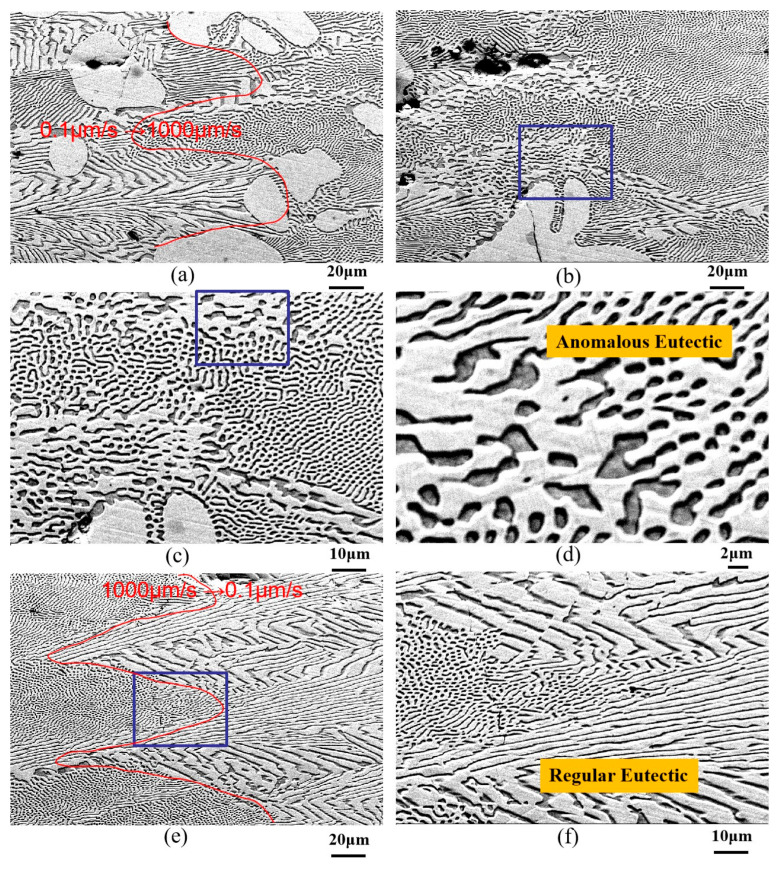
Microstructure evolution of Ni-33 wt.%Sn hypereutectic alloy during directional solidification with transition variable speed between 0.1 μm/s and 1000 μm/s: (**a**,**e**) are the transition interfaces (marked by red lines); (**b**) transition zone; (**c**,**d**,**f**) are the higher magnification images of rectangular regions shown in Figure 6b,c,e respectively.

Figure 6 shows microstructure evolution of Ni-33 wt.%Sn hypereutectic alloy during directional solidification with a transition variable speed between 0.1 μm/s and 1000 μm/s. As can be seen in Figure 6a,e, the transition interfaces can be divided into two parts based on the different microstructural morphology (marked by red lines). Figure 6a reveals that in the initial drawing speed of 0.1 μm/s, the white primary Ni_3_Sn phase is nearly cellular. The regions between primary dendrites on the left side of the interface are primarily filled with regular lamellar eutectic. Following the jump interface (right side), a higher proportion of rod eutectic is present. Figure 6b shows that with increasing jump magnitude, a small amount of anomalous eutectic forms at the interface (higher magnification in Figure 6c,d), surrounded by rod-like eutectic. The area fraction of anomalous eutectic is approximately 20% (Figure 6b). Subsequent jumps in drawing speed from 1000 μm/s to 0.1 μm/s (Figure 6e,f) lead to a transition back to regular lamellar/rod eutectic, but with coarser lamellar spacing. This coarsening suggests a jump to a lower drawing speed at this interface. With a further jump (presumably back to a higher rate), the coarse lamellar eutectic refines again. Additionally, under a steady-state drawing speed of 1000 μm/s, the primary Ni_3_Sn phase adopts an axial linear form, giving rise to a feathery eutectic structure. As for the Ni-33 wt.%Sn hypereutectic alloy, anomalous eutectic is not observed at all jump transition interfaces, indicating that its formation occurs within a specific processing window.

It can be clearly seen from Figure 6a,e that the directionally solidified Ni-Sn eutectic exhibits cellular eutectic growth at the interface front due to interface instability. Additionally, when the drawing speed increases from 0.1 μm/s to 1000 μm/s, the eutectic transforms from lamellar to rod-like; when the drawing speed decreases from 1000 μm/s to 0.1 μm/s, the eutectic morphology transforms from rod-like eutectic to lamellar eutectic. Melis et al. [5] present an in situ experimental study of the lamellar–rod morphological transition during directional solidification of a model eutectic transparent alloy with a drawing speed from 3.5 to 70.0 nms^−1^. They found that at a relatively large velocity, rod-like patterns formed systematically. In contrast, stable lamellae were observed at a sufficiently low velocity. Our experimental results are in good agreement with this result. The lamellar–rod morphological transition is a discontinuous process, and gives rise to highly nonlinear, particularly complex, spatio-temporal phenomena. The rod elongation instability governs the rod–lamellar transformation. Complex spatio-temporal phenomena involving labyrinth, oscillatory, and hybrid patterns were also reported, the multiscale dynamics of which are reminiscent of previous observations in directionally solidified eutectic [5].

As can be seen from Figure 4d, Figure 5d and Figure 6d, the anomalous eutectic coexists with the rod-like eutectic with a complex morphology. From our experiments, it can be found that the eutectic morphology undergoes a transition following the sequence “regular coarse lamellar eutectic → anomalous eutectic + rod-like eutectic → regular coarse lamellar eutectic” with the abrupt change in the drawing speed of the directional solidified Ni-33 wt.%Sn hypereutectic alloy.

In fact, Pu et al. [42] observed in the undercooled solidification of an Ni-P alloy that when the undercooling exceeds 190 K, the Ni-P eutectic morphology transforms from a regular (rod) structure to an anomalous eutectic; however, when the undercooling exceeds 300 K, the anomalous eutectic reverts back to cellular eutectic regions (regular lamellar/rod eutectic). That is, with increasing undercooling, the solidified microstructure undergoes a morphological change following the sequence “regular eutectic → anomalous eutectic → regular eutectic”. It can be found that the variation trend of eutectic morphology is relatively similar to that of the present experiment.

In our experiments, the anomalous eutectic always mixed with rod-like eutectic, which means the transition between regular eutectic to anomalous eutectic is also a complex spatio-temporal phenomena. Up to now, it remains controversial regarding the formation mechanism of anomalous eutectic.

### 3.3. CA Simulation of Ni-Sn Eutectic Microstructural Evolution During Directional Solidification

**Figure 7:**
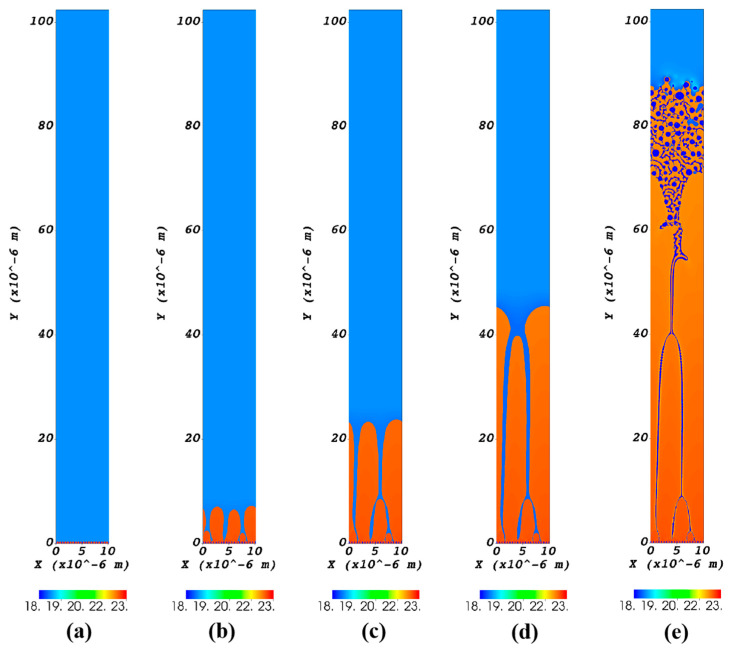
CA simulation of the eutectic transformation from regular eutectic to anomalous eutectic in directional solidification of Ni-Sn alloy (the drawing speed transitioned from 0.1 μm/s to 1000 μm/s within the first 0.06 s of time, where (**a**) t = 0.02 s; (**b**) t = 0.03 s; (**c**) t = 0.04 s; (**d**) t = 0.05 s; and (**e**) t = 0.06 s).

Figure 7 presents the simulated evolution of eutectic microstructure in Ni-32.5 wt.%Sn alloy during directional solidification with a transition variable speed, calculated using the cellular automaton (CA) method. The pulling velocity transitioned from 0.1 μm/s to 1000 μm/s within the first 0.06 s time. In Figure 7, orange regions represent the Ni_3_Sn phase, dark blue regions represent the α-Ni phase, and blue regions represent the liquid phase. As shown in Figure 7a, the microstructure is lamellar eutectic with a lamellar spacing of 0.5 μm at the first 0.02 s. From Figure 7b–d, the Ni_3_Sn phase epitaxially grows along the substrate in a cellular manner. The simulation clearly demonstrates that the jump in drawing speed triggers a morphological transition from regular lamellar eutectic to anomalous eutectic, as can be seen in Figure 7e. Furthermore, the CA results indicate that the rapid velocity jump destabilizes the regular lamellar eutectic. This instability allows the Ni_3_Sn phase to grow epitaxially along the substrate in a cellular manner, then the α-Ni phase to nucleates freely within the liquid ahead of the interface, which is followed by enveloping growth of the Ni_3_Sn phase around the α-Ni, leading to the formation of anomalous eutectic structures, which has been elaborated on in the reference [29].

**Figure 8:**
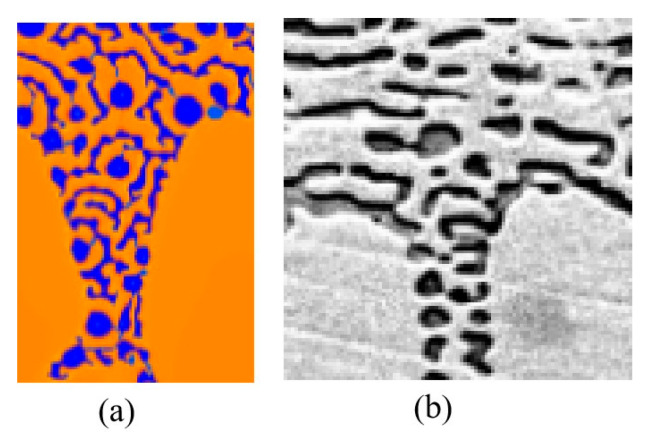
The comparison between the CA simulation and the experiments in detail, where (**a**,**b**) are the locally enlarged images of Figure 7e and Figure 6c, respectively.

Figure 8 is a detailed comparison picture between the CA simulation and the directional solidification experiments, where Figure 8a,b are the locally enlarged images of Figure 7e and Figure 6c, respectively. Interestingly, Figure 7e is very similar to Figure 6c in terms of details. The maximum size of α-Ni particles in CA simulation is approximately 1.3 μm, while that measured experimentally is approximately 3 μm, showing a difference in the microstructural characteristic scale, which may be due to the CA simulation neglecting thermal noise and simplifying diffusion [24]. In addition, 2D simulations limits the understanding of 3D eutectic growth morphologies. The computational domain is still not enough to capture the full experimental conditions.

It should be noted that although anomalous eutectic was observed in Ni-Sn eutectic alloys of all three compositions during directional solidification with a transition variable speed, their formation mechanisms differ. For the Ni-30 wt.%Sn hypoeutectic alloy and Ni-32.5 wt.%Sn eutectic alloy, anomalous eutectic formed under deceleration conditions (from 1000 to 0.1 μm/s), whereas for the Ni-33 wt.%Sn hypereutectic alloy, they emerged during acceleration stages (from 0.1 to 1000 μm/s). CA simulations of the acceleration process (from 0.1 to 1000 μm/s) have shown results remarkably similar to the experimental findings for the Ni-33 wt.%Sn hypereutectic alloy. That is to say, the CA simulation results show good agreement with the experimental observations of the Ni-33 wt.%Sn hypereutectic alloy from Bridgman directional solidification with a transition variable speed.

From our experimental results, when the Ni-Sn alloy is directionally solidified at a constant rate, it is not easy to produce anomalous eutectic structure; when the Ni-Sn alloy is directionally solidified at a variable speed, it is easy to produce anomalous eutectic structure similar to that under deep undercooling conditions. The rapid transition of growth rate will cause instability in the regular lamellar eutectic. From our CA simulation, the α-Ni phase and the Ni_3_Sn phase decouple growth and form anomalous eutectic. The Ni_3_Sn phase grows epitaxially along the substrate in a cellular morphology. Then the α-Ni phase nucleates randomly in the liquid phase at the front of the solid–liquid interface. At last, the Ni_3_Sn phase wraps the α-Ni phase to form anomalous eutectic.

During the process of solidification, the solute is continuously expelled from the solid–liquid interface front, forming a solute-rich layer at the interface front. When the liquidus in the enriched layer is lower than the actual temperature of the system, a composition undercooling region appears, which not only leads to destabilization of the flat interface, but may also lead to nucleation of a new phase [43,44]. From our CA simulation, it can be observed that when the Ni_3_Sn phase grows in cellular morphology, the nucleation of the α-Ni phase occurs at the front of the solid–liquid interface, and this should be the formation of anomalous eutectic.

### 3.4. The Relationship Among Growth Rate, Eutectic Microstructure, and Microhardness of Directionally Solidified Ni-Sn Alloys

Table 7 shows the lamellar spacing and microhardness of Ni-Sn alloy eutectic at different drawing speeds. From the Table 7, it can be observed that for a fixed alloy composition (e.g., Ni-33 wt.%Sn hypereutectic), as the drawing speed increases, the lamellar spacing gradually decreases, while the microhardness gradually increases. When the drawing speed is fixed (e.g., 1000 μm/s), the lamellar spacing of the Ni-32.5 wt.%Sn eutectic is very close to that of the interdendritic eutectic lamellae in the Ni-30 wt.%Sn hypoeutectic, and their microhardness values are comparable. This indicates that the composition has minimal impact on the Ni-Sn eutectic microstructure, which is primarily governed by the drawing speed.

**Figure 9:**
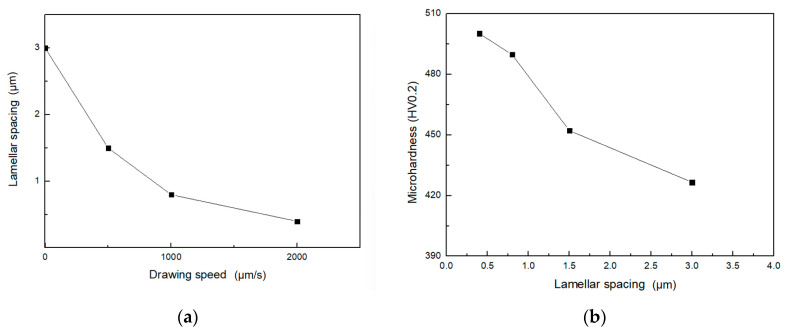
Dependence of lamellar spacing on (**a**) drawing speed and (**b**) microhardness for the directionally solidified Ni-Sn alloys.

Figure 9 presents the dependence of lamellar spacing on drawing speed Figure 9a and microhardness Figure 9b for the directionally solidified Ni-Sn alloys.

The relationship between eutectic lamellar spacing and microhardness can be described by the Hall–Petch formula [45]:

(4) $$HV=HV_{0}+K\lambda^{-0.5}$$

where *HV*_0_ is the initial hardness, and *K* is a constant.

According to the Hall–Petch relationship, the smaller the lamellar spacing, the higher the hardness of the material. A smaller lamellar spacing means a shorter free path for dislocation movement, and dislocations are more likely to be blocked by the phase interface. The lamellar eutectic structure is formed by the alternate arrangement of two different phases in a lamellar pattern, resulting in a large interfacial area between the phases. When dislocations encounter the phase interface during their movement, they are strongly impeded. This is because the atomic arrangement and crystal structure on both sides of the phase interface are different, making it difficult for dislocations to directly cross the phase interface. This is the phase-boundary strengthening mechanism [45]. As can be seen from Table 7, for the Ni-32.5 wt.%Sn eutectic, when the pulling rate is 2000 μm/s, the eutectic lamellar spacing is the smallest, at approximately 0.4 μm, and the microhardness of this structure is the largest, at approximately 500 HV.

In addition, hardness tests were also conducted on rod-like eutectic and anomalous eutectic. It was found that the average hardness of rod-like eutectic was 515.9 HV0.2, while that of anomalous eutectic was 406.9 HV. Under directional solidification conditions, lamellar eutectic and rod-like eutectic have higher hardness, while anomalous eutectic has lower hardness.

Wang et al. [46] tested the anomalous eutectic at the bottom of the sample when studying the laser-melted Ni-33 wt.%Sn hypereutectic alloy. They found the microhardness of anomalous eutectic is approximately 370 HV. The anomalous eutectic structure under directional solidification conditions is finer compared with that at the bottom of the laser melted sample, thus exhibiting higher hardness.

Yan et al. [47] undercooled CoCrFeNiMo0.8 high entropy alloy (HEA) up to 205 K with an electromagnetic levitation technique. A kinetics transition from lamellar eutectic to anomalous eutectic was observed. With the increase in undercooling, the hardness, strength and compression of this HEA are enhanced to different extents. They pointed out that the plasticity improvement may result from the volume fraction increase in anomalous eutectic. Because anomalous eutectic exhibits a better plastic deformation capacity through structural modulation, it is beneficial to design HEAs with superior properties.

Regarding the industrial applications of anomalous eutectic, in addition to its application in eutectic high entropy alloys [47], it may be also used in the research of solder alloys. As for the Sn-Pb eutectic solder alloy, it is found that the microstructure morphology of Sn37Pb is very similar to that of anomalous eutectic [48]. Moreover, it is speculated that anomalous eutectic microstructure is related to the soldering performance of some solders, which can be further studied with respect to control of anomalous eutectic in solder alloys.

## 4. Conclusions

Bridgman directional solidification experiments conducted within a drawing speed range of 0.1–2000 μm/s showed that constant drawing speeds consistently produced regular lamellar/rod-like microstructures. Anomalous Ni-Sn eutectic structures, resembling those formed under deep undercooling conditions, were identified specifically within transition zones associated with abrupt changes (jumps between 0.1 and 1000 μm/s) in drawing speed.

(1) The novelty of the velocity-jump methodology includes being able to achieve different growth conditions by varying speed in the same sample and more accurately comparing the structures under different conditions; enabling the study of dynamic change processes, such as the transformation mechanism of morphology during speed variation; precisely controlling parameters to investigate critical conditions for lamellar/rod–anomalous eutectic transition (ΔV = 1000 μm/s); and potentially observing unsteady-state growth behaviors, which enriches the theory of eutectic growth.
(2) The CA simulations revealed that rapid jumps in growth velocity destabilize the regular lamellar eutectic. This instability facilitates the epitaxial Ni_3_Sn phase and independent α-Ni nucleation, ultimately forming the anomalous eutectic structure. The α-Ni phase and the Ni_3_Sn phase exhibit decoupled growth to form anomalous eutectic. The CA simulation results are in good agreement with the experimental findings from Bridgman directional solidification.
(3) In this work, as the drawing speed increased, the lamellar spacing decreased, while the microhardness increased and followed the Hall–Petch relationship. The microhardness of anomalous eutectic is lower than lamellar/rod-like eutectic. However, anomalous eutectic exhibits a better plastic deformation capacity through structural modulation in high entropy alloy [47], which is beneficial to design multi-component alloys with superior properties. Regarding the lamellar–rod transition mechanism of eutectic, some scholars have adopted transparent system simulation and in situ observation technology [5], and this method can also be used to investigate the formation mechanism of anomalous eutectic in future work.

## Figures and Tables

**Table 1:** ICP analysis results of impurity elements in Ni blocks.

Impurity Elements	Fe	Cu	Co	Pb	Zn	Cr	Mg	Al	Bal.	Total
Measured values (mg/kg)	8.4	3.3	2.2	1.4	5.3	2.9	3.6	5.2	<12.0	44.3
Standard requirements (<mg/kg)	50	30	20	10	20	20	30	50	<100	<100

**Table 2:** ICP analysis results of impurity elements in Sn particles.

Impurity Elements	Fe	Cu	Pb	Sb	As	Bi	Zn	Bal.	Total
Measured values (mg/kg)	10.2	4.9	2.7	3.3	1.1	0.9	6.2	<15.3	52.1
Standard requirements (<mg/kg)	50	30	20	20	10	10	30	<100	<100

**Table 3:** EDS results for the composition of three Ni-Sn as-cast ingots.

Composition	Ni-30 wt.%Sn Ingot	Ni-32.5 wt.%Sn Ingot	Ni-33 wt.%Sn Ingot
Ni (wt.%)	69.11	67.66	66.38
Sn (wt.%)	30.89	32.24	33.62

**Table 4:** Experiment scheme for Ni-32.5 wt.%Sn eutectic alloy by Bridgman directional solidification.

Segment Number	Starting Speed (μm/s)	Termination Speed (μm/s)	Time (s)
1	2000.0	2000.0	5
2	0.1	2000.0	1
3	2000.0	2000.0	5
4	0.1	2000.0	3
5	2000.0	2000.0	5
6	0.1	2000.0	5
7	2000.0	2000.0	5
8	0.1	2000.0	7
9	2000.0	2000.0	20

**Table 5:** Experiment scheme for Ni-Sn alloys by Bridgman directional solidification.

Segment Number	Starting Speed (μm/s)	Termination Speed (μm/s)	Time (s)
1	1000.0	1000.0	25
2	0.1	1000.0	10
3	1000.0	1000.0	5
4	0.1	1000.0	20
5	1000.0	1000.0	5
6	0.1	1000.0	30
7	1000.0	1000.0	30

**Table 6:** The average lamellar spacing and Ni_3_Sn phase size under different drawing speeds of Ni-33 wt.%Sn alloy.

Drawing Speed (μm/s)	Average Spacing of Lamellar Eutectic (μm)	Average Width of Primary Ni_3_Sn Phase (μm)
0.1	3	28
500	1.5	20
1000	0.5	7

**Table 7:** The lamellar spacing and microhardness of Ni-Sn alloys under different drawing speeds.

Composition of Alloy	Drawing Speed (μm/s)	Spacing of Lamellar Eutectic (μm)	Vickers Microhardness (HV 0.2)
Ni-30 wt.%Sn hypoeutectic	0.1	1.78~2.12	436.9
Ni-30 wt.%Sn hypoeutectic	1000	0.76~0.81	470.5
Ni-32.5 wt.%Sn eutectic	1000	0.87~0.91	473.2
Ni-32.5 wt.%Sn eutectic	2000	0.41~0.47	500.2
Ni-33 wt.%Sn hypereutectic	0.1	2.45~3.02	426.6
Ni-33 wt.%Sn hypereutectic	500	1.39~1.51	452.2
Ni-33 wt.%Sn hypereutectic	1000	0.54~0.78	489.8

## Data Availability

The original contributions presented in this study are included in the article. Further inquiries can be directed to the corresponding authors.

## References

[1] Chao P., Aramanda S.K., Xiao X., Bottin-Rousseau S.B., Akamatsu S., Shahani A.J. (2024). From Irregular to Regular Eutectic Growth in the Al-Al_3_Ni system: In Situ Observations During Directional Solidification. Acta Mater..

[2] Wang X., Zhai W., Li H., Wang J.Y., Wei B. (2023). Ultrasounds induced eutectic structure transition and associated mechanical property enhancement of FeCoCrNi_2.1_Al high entropy alloy. Acta Mater..

[3] Jin G., Liu Z., Wang Y., Yu L., Li S., Xing H. (2025). Formation of Seaweed Morphology and Enhancing Mechanical Properties of an A356 Alloy by Directional Solidification. J. Alloys Compd..

[4] Zhao K., Wu S., Jiang S., Zhang H., Song K., Wang T., Xing H., Zhang L., Li K., Yang L. (2020). Microstructural Refinement and Anomalous Eutectic Structure Induced by Containerless Solidification for High-Entropy Fe–Co–Ni–Si–B Alloys. Intermetallics.

[5] Melis S., Sabine B.R., Silvère A. (2023). Lamella-Rod Pattern Transition and Confinement Effects During Eutectic Growth. Acta Mater..

[6] Dong H., Chen Y.Z., Zhang Z.R., Shan G.B., Zhang W.X., Liu F. (2020). Mechanisms of Eutectic Lamellar Destabilization upon Rapid Solidification of an Undercooled Ag-39.9 at.% Cu Eutectic Alloy. J. Mater. Sci. Technol..

[7] Fiore G., Quaglia A., Battezzati L. (2019). Banded Regular/Anomalous Eutectic in Rapidly Solidified Co-61.8 at.% Si. Scr. Mater..

[8] Trivedi R., Magnin P., Kurz W. (1987). Theory of eutectic growth under rapid solidification conditions. Acta Metall..

[9] Mullis A.M., Clopet C.R. (2018). On the Origin of Anomalous Eutectic Growth From Undercooled Melts: Why Re-melting is not a Plausible Explanation. Acta Mater..

[10] Zhang F., Zhang J., Lü X., Hua K., Zhao Y., Wang H. (2024). Revealing the nucleation and growth modes upon rapid solidification of undercooled Co-24 at.% Sn eutectic alloy by the crystallographic orientation relations. J. Alloys Compd..

[11] Soodabeh A., Jeffrey E.S. (2020). Multiple origins of anomalous eutectic microstructure in rapidly solidified Mg-Al alloy. Materialia.

[12] Li M., Kuribayashi K. (2003). Nucleation-controlled microstructures and anomalous eutectic formation in undercooled Co-Sn and Ni-Si eutectic melts. Metall. Mater. Trans. A.

[13] Zhang F., Zhang J., Lü X., Hua K., Zhao Y., Wang H. (2022). Crystallographic evidences for twin-assisted eutectic growth in undercooled Ni-18.7 at.% Sn eutectic melts. J. Mater. Sci. Technol..

[14] Dong H., Chen Y.Z., Wang K., Shan G.B., Zhang Z.R., Zhang W.X., Liu F. (2020). Modeling Remelting Induced Destabilization of Lamellar Eutectic Structure in an Undercooled Ni-18.7 at.% Sn Eutectic Alloy. J. Alloys Compd..

[15] Zhao R., Wang Y., Gao J., Baker E.B., Matson D.M., Kolbe M., Chuang A.C.P., Ren Y. (2020). In Situ and Ex Situ Studies of Anomalous Eutectic Formation in Undercooled Ni–Sn Alloys. Acta Mater..

[16] Fu Y., Guo X., Xiao Z. (2025). Microstructure Evolution upon Directional Solidification Process of Nb-Si Based Ultrahigh Temperature Alloy. J. Alloys Compd..

[17] Li M., Qi Z., Peng H., Hou R., Chen Y., Zheng G. (2025). Lamellar Orientation Modulation of Fe-Al Alloys. Mat. Sci. Eng. A.

[18] Hao Y., Xu X., Wu Q., Wu L., Zhao Y., Hou H. (2023). Investigation on Nonequilibrium Crystallization of Highly Undercooled Cu–Ni–Co Alloys. J. Mater. Res. Technol..

[19] Wang N., Jia L., Kong B., Guo Y., Zhang H., Zhang H. (2018). Eutectic Evolution of Directionally Solidified Nb-Si Based Ultrahigh Temperature Alloys. Int. J. Refract. Met. Hard Mater..

[20] Son H.Y., Jung I.Y., Choi B.G., Shin J.H., Jo C.Y., Lee J.H. (2024). Effects of Chemical Composition and Solidification Rate on the Solidification Behavior of High-Cr White Irons. Metals.

[21] Cui C., Wang C., Wang P., Liu W., Lai Y., Deng L., Su H. (2020). Microstructure and Fracture Toughness of the Bridgman Directionally Solidified Fe-Al-Ta Eutectic at Different Solidification Rates. J. Mater. Sci. Technol..

[22] Kang J., Li J. (2021). Microstructural Evolution in Directional Solidification of Nb-doped Co-Sn/Ni-Sn Eutectic Alloys. Appl. Phys. A.

[23] Campo K.N., Wischi M., Rodrigues J.F.Q., Starck L.F., Sangali M.C., Caram R. (2024). Directional Solidification of the Al_0.8_CrFeNi_2.2_ Eutectic High-Entropy Alloy. J. Mater. Res. Technol..

[24] Wei L., Cao Y., Lin X., Wang M., Huang W. (2019). Quantitative Cellular Automaton Model and Simulations of Dendritic and Anomalous Eutectic Growth. Comput. Mater. Sci..

[25] Clopet C.R., Cochrane R.F., Mullis A.M. (2013). Spasmodic Growth During the Rapid Solidification of Undercooled Ag-Cu Eutectic Melts. Appl. Phys. Lett..

[26] Lin X., Cao Y., Wang Z., Cao J., Wang L., Huang W. (2017). Regular eutectic and anomalous eutectic growth behavior in laser remelting of Ni-30wt.%Sn alloys. Acta Mater..

[27] Zhao R., Gao J., Liao H., Fenineche N., Coddet C. (2020). Selective laser melting of elemental powder blends for fabrication of homogeneous bulk material of near-eutectic Ni-Sn composition. Addit. Manuf..

[28] Requena G., Bugelnig K., Sket F., Milenkovic S., Rödler G., Weisheit A., Gussone J., Haubrich J., Barriobero-Vila P., Pusztai T. (2020). Ultrafine Fe-Fe_2_Ti eutectics by directed energy deposition: Insights into microstructure formation based on experimental techniques and phase field modelling. Addit. Manuf..

[29] Wei L., Cao Y., Lin X., Huang W. (2018). Cellular Automaton Simulation of the Growth of Anomalous Eutectic during Laser Remelting Process. Materials..

[30] Kurz W., Fisher D.J., Rappaz M. (2023). Fundamentals of Solidification.

[31] Ma X., Liu L. (2015). Solidification Microstructures of the Undercooled Co-24at%Sn Eutectic Alloy Containing 0.5at%Mn. Mater. Des..

[32] Yamauchi I., Ueyama S., Ohnaka I. (1996). Effects of Mn and Co Addition on Morphology of Unidirectionally Solidified FeSi_2_ Eutectic Alloys. Mater. Sci. Eng. A.

[33] Zhao S., Li J., Liu L., Zhou Y. (2009). Eutectic growth from cellular to dendritic form in the undercooled Ag-Cu eutectic alloy melt. J. Cryst. Growth.

[34] Tang P., Tian Y., Liu S., Lv Y., Xie Y., Yan J., Liu T., Wang Q. (2021). Microstructure Development in Eutectic Al-Fe Alloy During Directional Solidification under High Magnetic Fields at Different Growth Velocities. J. Mater. Sci..

[35] Wei B., Yang G., Zhou Y. (1991). High Undercooling and Rapid Solidification of Ni-32.5%Sn Eutectic Alloy. Acta Metall. Mater..

[36] Jackson K.A., Hunt J.D. (1966). Lamellar and Rod Eutectic Growth. Trans. Metall. Soc. AIME..

[37] Qin Q., Yang L., Li J. (2025). Halo Formation in Solidification of Off-eutectic Alloys. Metall. Mater. Trans. A.

[38] Qin Q.Y., Li J.F., Yang L., Liu L.J. (2024). Solidification Behavior and Microstructure of Ag–Cu Eutectic Alloy at Different Sub-Rapid Cooling Rates. Mater. Chem. Phys..

[39] Karma A. (1987). Beyond Steady-State Lamellar Eutectic Growth. Phys. Rev. Lett..

[40] Lin X., Huang W.D., Fen J. (1999). History dependent selection of primary cellular dendritic spacing during unidirectional solidification in aluminum alloys. Acta Mater..

[41] Liu L.J., Wei X.X., Ferry M., Li J.F. (2020). Investigation of the Origin of Anomalous Eutectic Formation by Remelting Thin-Gauge Samples of an Ag-Cu Eutectic Alloy. Scr. Mater..

[42] Pu J., Feng W.J., Xiao J.Z., Gan Z.H., Yi H.Y., Cui K. (2003). Non-equilibrium solidification of bulk undercooled Ni-P eutectic alloys. J. Cryst. Growth.

[43] Li J., Qiao D., Dong S., Peng P., Yan X., Zhang X. (2023). Analysis on phase selection and microstructure evolution in directionally solidified Zn-Al-Mg-Ce alloy. China Foundry.

[44] Trived R., Kurz W. (1990). Modeling of solidification microstructures in concentrated solutions and intermetallic systems. Metall. Mater. Trans. A.

[45] Wischi M., Campo K.N., Starck L.F., Fonseca E.B., Lopes É.S.N., Caram R. (2022). Microstructure and mechanical behavior of the directionally solidified AlCoCrFeNi_2.1_ eutectic high-entropy alloy. J. Mater. Res. Technol..

[46] Wang Z., Lin X., Cao Y., Huang W. (2013). Microstructure evolution in laser surface remelting of Ni–33wt.% Sn alloy. J. Alloys Compd..

[47] Yan P.X., Chang J., Wang W.L., Zhu X.N., Lin M.J., Wei B. (2022). Eutectic growth kinetics and microscopic mechanical properties of rapidly solidified CoCrFeNiMo_0.8_ high entropy alloy. Acta Mater..

[48] An Q., Zhang M., Wang N., Fei J. (2025). Effects of Interaction Between Sn-Rich and Pb-Rich Phases on the Mechanical Properties of Sn-Pb Eutectic Solder Alloy at Cryogenic Temperature. J. Electron. Mater..

